# The ecological security risks of bronopol: a focus on antibiotic resistance gene dissemination

**DOI:** 10.3389/fmicb.2025.1595833

**Published:** 2025-07-07

**Authors:** Zhuocheng Yao, Yuhan Yang, Yanchun Gong, Shiyi Shi, Yunying Ge, Weiliang Zeng, Deyi Zhao, Jianming Cao, Tieli Zhou, Mo Shen

**Affiliations:** ^1^Key Laboratory of Clinical Laboratory Diagnosis and Translational Research of Zhejiang Province, Department of Clinical Laboratory, The First Affiliated Hospital of Wenzhou Medical University, Wenzhou, China; ^2^School of Laboratory Medicine and Life Science, Wenzhou Medical University, Wenzhou, China

**Keywords:** bronopol, horizontal gene transfer, RP4-7, *bla*_NDM-4_, disinfectant

## Abstract

Disinfectants are commonly utilized by humans to combat microorganisms. However, residual disinfectants may promote environmental antimicrobial resistance by facilitating horizontal gene transfer (HGT) of antibiotic resistance genes. Bronopol is a routinely used disinfectant that persists in the environment, and previous studies have concentrated on its ecotoxicity rather than its implications on the propagation of resistance genes. This study aimed to establish an *in vitro* conjugation model to investigate whether bronopol promotes the transfer of antibiotic resistance genes (ARGs) via plasmid conjugation. Using *Escherichia coli* DH5α and DC8855 as donors harboring RP4-7 and *bla*_NDM-4_-positive *IncFII(K)* plasmids, respectively, and J53 as the recipient strain, we found that sub-inhibitory concentrations of bronopol (2 μg/L and 20 μg/L) significantly increased the conjugative transfer frequency (CTF) of both plasmids. Mechanistic analysis revealed that bronopol enhanced bacterial membrane permeability, as demonstrated by propidium iodide (PI) staining, 1-N-phenylnaphthylamine (NPN) fluorescent probes, transmission electron microscopy (TEM), and upregulation of the outer membrane protein gene *ompC*. Additionally, bronopol treatment upregulated RP4 plasmid-encoded genes involved in DNA transfer/replication (*trfAp*) and the global regulator of HGT (*kilA*/*kilB*). These findings highlight a previously unrecognized role of bronopol in facilitating the dissemination of antibiotic resistance genes, particularly those of clinical significance.

## 1 Introduction

Antimicrobial resistance (AMR) is mostly attributable to the extensive utilization of antibiotics in agriculture and healthcare, resulting in the rapid emergence of antibiotic-resistant bacteria (Aminov, 2009). AMR is expected to cause 10 million deaths by 2050, with a total economic cost of $100 trillion (Patricios et al., 2023). In addition to the unavoidable establishment of medication resistance, the fast spread of drug-resistant genes is especially concerning in the long run. Mutation, vertical gene transfer (VGT), and horizontal gene transfer (HGT) are the primary mechanisms by which AMR is transmitted (Neil et al., 2021; Guzman-Otazo et al., 2022).

Antibiotic-resistant genes (ARGs) are transmitted in the environment mostly by HGT (Woods et al., 2020; Xu et al., 2021). There are three major HGT pathways: transformation, transduction, and conjugation (Phan et al., 2024). Conjugation is the most common HGT mechanism, involving direct physical contact between cells via columnar bridges or pore channels (Meng et al., 2022; Zhuang et al., 2024). When bacteria are exposed to severe environmental conditions, such as the presence of antimicrobial drugs, they may acquire ARGs, which allow them to change their genomes for increased flexibility and adaptation (Liu et al., 2019; Song et al., 2020). Notably, the frequency of conjugation among bacteria remains modest, although certain foreign chemicals have the ability to accelerate this process. For example, the antibacterial medication mucin (Xiao et al., 2022), the antiepileptic drug carbamazepine (Wang et al., 2019), non-nutritive sweeteners (Yu et al., 2021), and disinfectants such as triclosan and hydrogen peroxide (Lu et al., 2018) have been shown to favor the conjugation process.

Bronopol (2-bromo-2-nitro-1,3-propanediol) is a preservative and broad-spectrum biocide widely used in food industry, cosmetics formulation, and aquaculture product development (Peters et al., 1983; Butler and Stergiadis, 2011; Carbajo et al., 2015; Lopez-Sanchez et al., 2021; Wang et al., 2022). While existing research has primarily focused on its environmental and human health risks (Perrenoud et al., 1994; Aerts et al., 2020; Lopez-Sanchez et al., 2021; Magara et al., 2021; Wang et al., 2023), the role of bronopol in plasmid-mediated conjugative transfer of ARGs remains unexplored (Vijayakumar and Sandle, 2019). *Escherichia coli* (*E. coli*) serves as a universal model for studying intraspecific conjugative transfer (Zhu et al., 2023; Yang et al., 2024). To gain fundamental insights into bronopol-bacteria interactions and their potential clinical hazards, this study investigated the impact of bronopol on conjugative transfer of the RP4-7 and *bla*_NDM-4_-positive *IncFII(K)* plasmids in *E. coli* strains, exploring underlying mechanisms involving membrane permeability, reactive oxygen species (ROS) production, and conjugation-related genes expression. Our findings reveal that bronopol promotes conjugative transfer, representing a previously unrecognized pathway for environmental dissemination of ARGs.

## 2 Materials and methods

### 2.1 Bacterial strains and disinfectants

*Escherichia coli* DH5α, which possesses the RP4-7 plasmid containing chloramphenicol and ampicillin resistance genes, acted as the donor, while *E. coli* J53, resistant to sodium azide, acted as the recipient. Both isolates were procured from the laboratory for conjugation testing. Bronopol originated from Aladdin, and phosphate-buffered saline (PBS) was employed as the solvent.

### 2.2 Proliferation of donor and recipient bacterial strains

Overnight cultures of J53 and DH5α were diluted in Luria-Bertani (LB) broth to a turbidity of 0.5 McFarland standard. Bronopol was administered to each bacterium to achieve final concentrations of 2 μg/L, 20 μg/L, and 200 μg/L. Samples devoid of bronopol served as controls. The mixes were incubated statically, and OD_600_ was recorded hourly for a duration of 16 h. Each group was examined three times.

### 2.3 *In vitro* conjugative transfer system

Conjugation experiments between donor and recipient bacteria were conducted under bronopol exposure using a modified protocol adapted from the previous study (Pallares-Vega et al., 2021). Bacteria were cultivated in LB broth at 37°C and harvested using centrifugation. A McFarland turbidity of 0.5 was then attained by resuspending the bacterial precipitate in PBS, resulting in a final bacterial density of 1.5 × 10^8^ CFU/mL, thereby forming the final conjugation system with a total volume of 2 mL. One milliliter of either donor or recipient bacteria was combined with varying concentrations of bronopol (2 μg/L and 20 μg/L), then incubated statically for 12 h at 37°C. Plates were prepared by adding 5 μL of conjugation mixture to LB agar supplemented with sodium azide (200 mg/L) and ampicillin (100 mg/L), then incubated under standard conditions. Transconjugant enumeration was performed by plating on LB agar medium containing 200 mg/L sodium azide to quantify recipient counts. CTF was calculated as the transconjugant-to-recipient ratio. Antimicrobial susceptibility tests verified representative colonies from conjugate crosses. All mating experiments were conducted in biological triplicate.

### 2.4 Reactive oxygen species (ROS) production and membrane permeability assay

Intracellular ROS levels were measured with a Cellular ROS Assay Kit (Beyotime, Shanghai, China) per the manufacturer’s protocol. Donor and recipient cultures were grown overnight to an OD_600_ of 0.5 and resuspended in PBS. Bacteria were incubated with 10 μM DCFH-DA at 37°C for 30 min in the dark. The unbound probe was removed via two PBS washes. After a 2-h incubation at 37°C, fluorescence intensity was recorded using Infinite M200 Microplate Reader at 488 nm excitation/525 nm emission. All experiments were performed in triplicate biological repeats. Membrane permeability was evaluated with 0.5 μM PI (propidium iodide) and 10 μM NPN (1-N-phenylnaphthylamine) (Beyotime). PI excitation/emission was 535 nm/615 nm, while NPN was 350 nm/420 nm. All tests were conducted in triplicate. According to the previous experimental protocol (Zhang et al., 2022), we analyzed bacterial membrane permeability using Confocal laser scanning microscopy (CLSM). Bacteria with a 0.5 McFarland turbidity were inoculated into PBS containing 2 μg/L bronopol or 20 μg/L bronopol and treated at 37°C for 12 h. The samples were then incubated at room temperature in PI (50 mg/L) for 20 min. Bright-field and fluorescent images were captured using CLSM (LSM800, Zeiss, Jena, Germany).

### 2.5 Analysis with TEM

Following 12-h exposure to 20 μg/L bronopol, bacterial cell ultrastructure was analyzed via transmission electron microscopy (TEM). *E. coli* DH5α and J53 cultures were harvested by centrifugation at 5,000 × *g* for 6 min, washed twice with ice-cold PBS, and resuspended in PBS. Cells were fixed in 2.5% (v/v) glutaraldehyde in 0.1 M sodium cacodylate buffer (pH 7.4) at 4°C overnight. Specimens were dehydrated through a graded ethanol series (50, 70, 90, 100% for 15 min each), infiltrated with Epon-Araldite epoxy resin, and polymerized at 60°C for 48 h. Ultrathin sections (70 nm) were prepared using an EM UC7 ultramicrotome (Leica, Germany), post-stained with 2% uranyl acetate and lead citrate, and imaged on a Tecnai T12 TEM (Thermo Fisher Scientific, USA) operated at 120 kV.

### 2.6 Expression levels of mRNA from conjugative transfer-related genes

Bacterial cultures (1.5 × 10^8^ CFU/mL) were treated with 20 μg/L bronopol at 37°C for 12 h. Total RNA was extracted using the EASYspin Bacterial RNeasy Mini Kit (Aidlab, China) as per the manufacturer’s instructions. cDNA was synthesized via reverse transcription with the PrimeScript RT reagent Kit (TaKaRa, Japan). qRT-PCR was carried out on an Applied Biosystems 7500 Fast Real-Time PCR System (Thermo Fisher Scientific, USA) using SYBR Premix Ex Taq II (TaKaRa, Japan). Relative mRNA levels were normalized to the 16S rRNA using the 2^−ΔΔ^Ct method. Primer sequences are in Supplementary Table S1. All experiments were done in triplicate.

### 2.7 Statistical analysis

GraphPad Prism version 8.2.1 was used to analyze the data. The mean ± standard deviation is used to display the data. Independent samples *t*-test was used to assess significant differences, and *p* < 0.05 was considered statistically significant.

## 3 Results

### 3.1 The minimum inhibitory concentration (MIC) of bronopol

**Figure 1:**
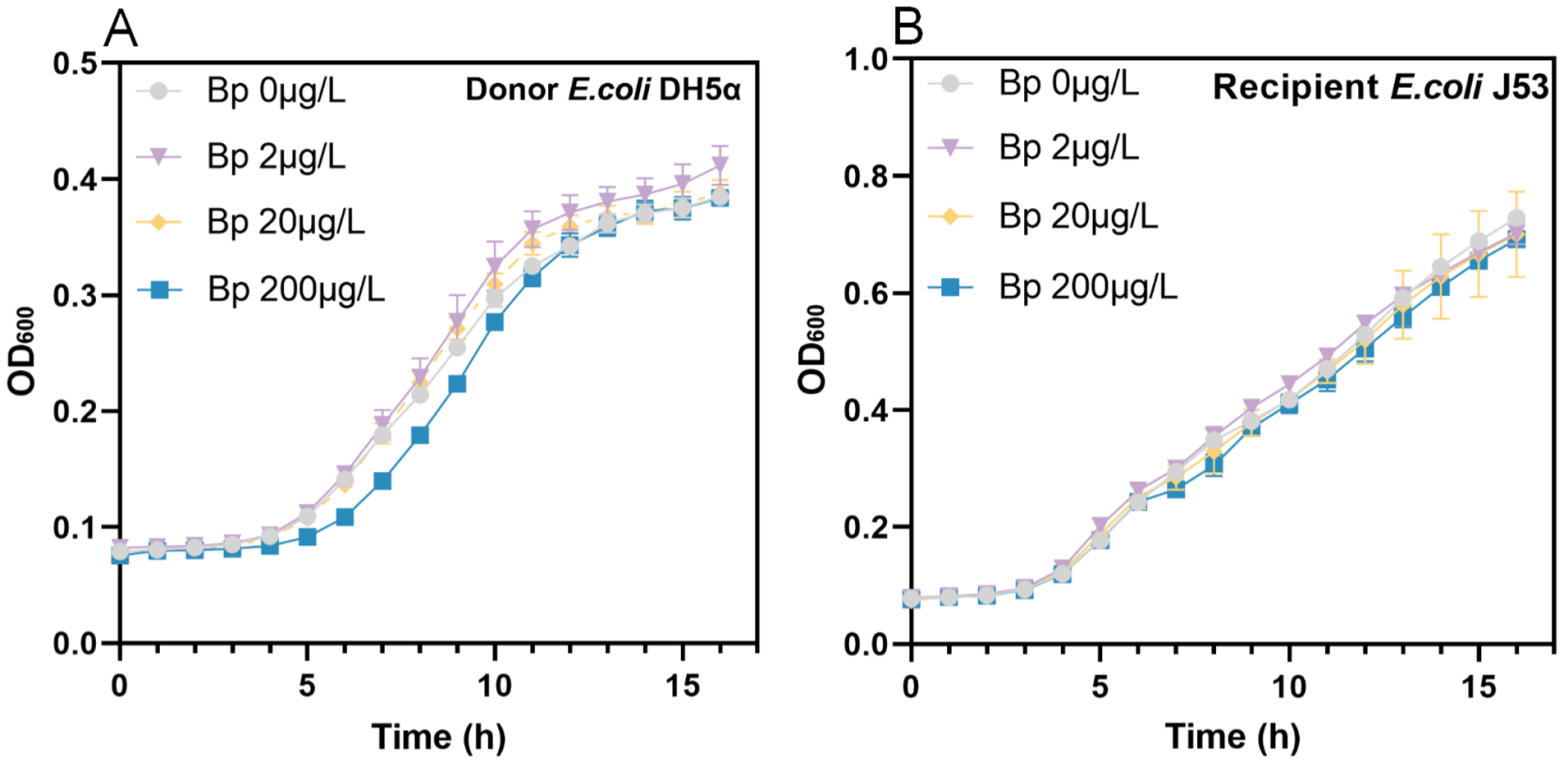
Growth curves of **(A)** donor (*E. coli* DH5α) and **(B)** recipient (*E. coli* J53) strains exposed to sub-inhibitory concentrations of bronopol.

Bronopol exhibited strain-specific minimum inhibitory concentrations (MICs), with values of 2 mg/L for DH5α and 4 mg/L for both J53 and DC8855 (Figure 1 and Table 1). After 24 h, all treatment groups with sub-inhibitory concentrations achieved growth levels comparable to the control group. However DH5α treated with 200 μg/L bronopol displayed a lower growth rate during the exponential phase (5–12 h post-treatment; Figure 1A). To ensure consistent bacterial growth, which is a critical requirement for comparing the changes in the conjugative transfer frequency, in the subsequent experiments, we selected the concentrations of 2 μg/L and 20 μg/L.

**Table 1:** Basic information of clinical strain used in the conjugation assay.

Strains	MLST types	Plasmid names	Resistance genes	Accession no. (NCBI)
DC8855	ST12531	pDC8855-NDM-4 【*IncFII(K)*】	*blaNDM-4*, *blaLAP-2*, *qnrS1, aac(3)-IId, blaCTX-M-14*	CP146021

### 3.2 The subinhibitory concentration of bronopol enhances the conjugative transfer frequency of plasmids

**Figure 2:**
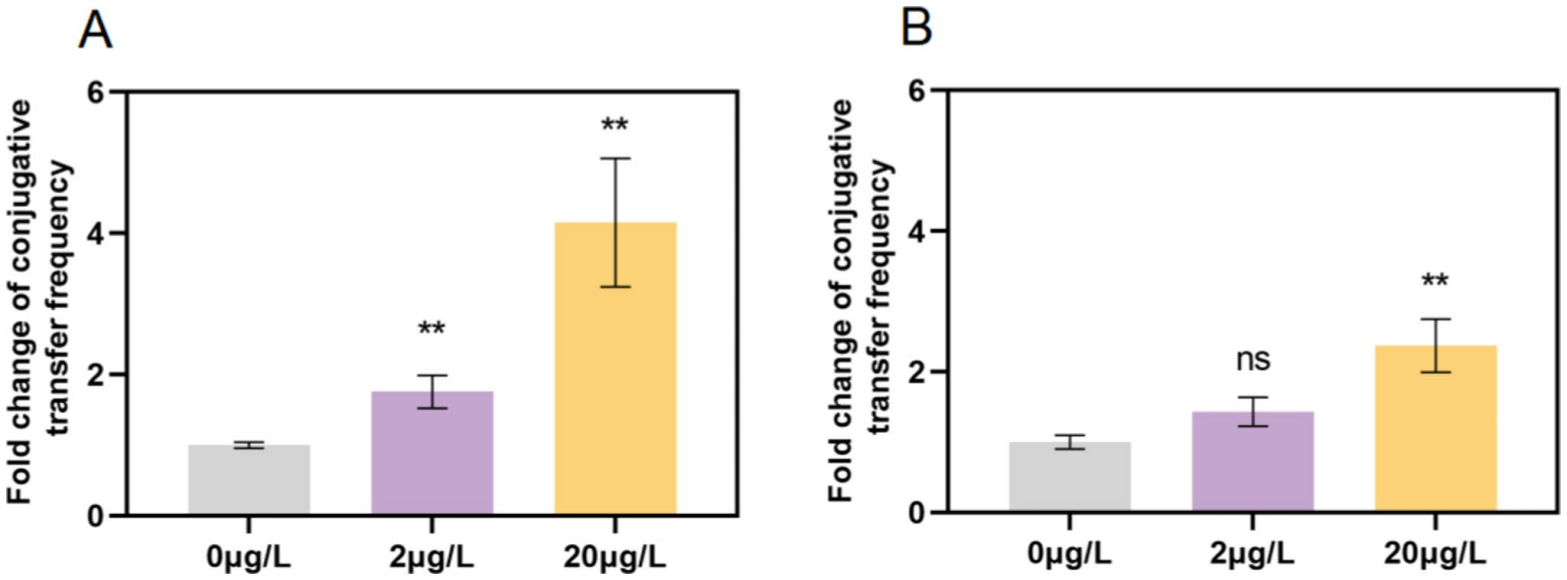
Bronopol increases the conjugation frequencies of the RP4-7 plasmid **(A)** and the *IncFII(K)* plasmid **(B)**. Independent-samples *t*-test was used to compare bronopol-treated groups to the blank control (drug-free): ns, not significant, **p < 0.01; ***p < 0.001.

Sub-inhibitory concentrations of bronopol significantly enhanced the conjugative transfer frequency (CTF) of the RP4-7 plasmid in a dose-dependent manner (Figure 2A). At 20 μg/L, the CTF increased by 4.15-fold (*p* < 0.01) compared to the control, while a 1.76-fold increase was observed at 2 μg/L (*p* < 0.05). To ascertain whether bronopol could enhance the CTF of clinically relevant wide-host-range plasmids and to explore its implications for the spread of clinically significant carbapenem-resistant genes, we concurrently measured the CTF of the *IncFII(K)* plasmid harboring the *bla*_NDM-4_ gene (Figure 2B). While bronopol induced a weaker CTF promotion for *IncFII(K)* plasmid (115,297 bp, >10 KB) compared to RP4-7 (60,002 bp), a 2.32-fold increase (*p* < 0.05) was detected for *IncFII(K)* at 20 μg/L. This indicates that bronopol at residual concentrations in the environment has a low capacity to increase the conjugative transfer frequency of large plasmids.

### 3.3 Impact of bronopol on cell membrane penetration

**Figure 3:**
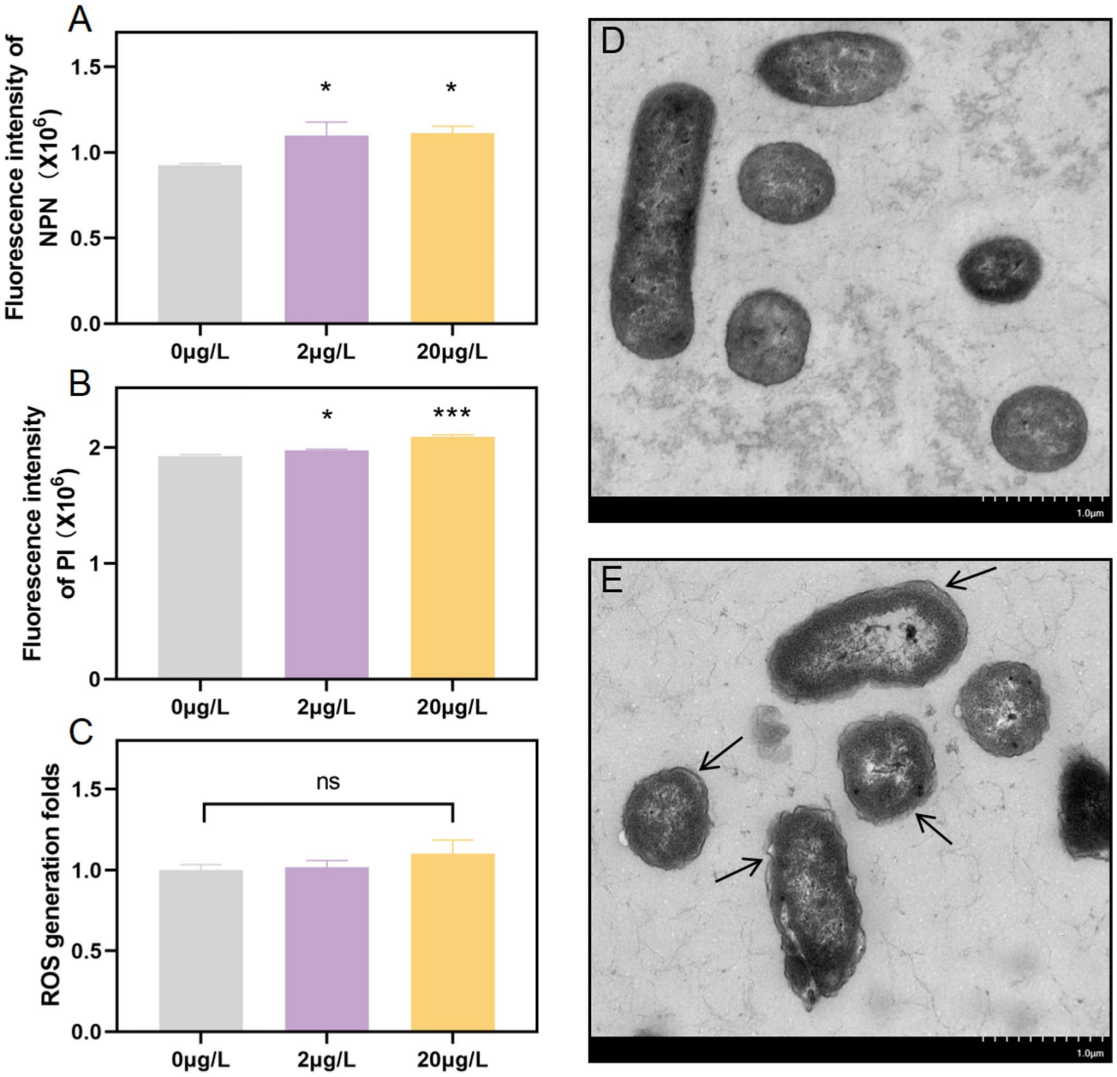
**(A)** Alterations in outer membrane permeability evaluated using the NPN probe after exposure to subinhibitory doses of bronopol. **(B)** Assessment of inner membrane permeability alterations using PI probe following exposure to subinhibitory doses of bronopol. **(C)** Variations in ROS production resulting from exposure to subinhibitory concentrations of bronopol. **(D)** Blank control treatment group. **(E)** Surface morphology was examined using scanning electron microscopy with 20 μg/L of bronopol. Substantial differences between the bronopol-treated groups and the bronopol 0 μg/L were established using one-way analysis of variance: *p < 0.05; ***p < 0.001; ns, not significant.

The cell membrane plays a pivotal role in the conjugation process. To delve into the biological mechanism of conjugative transfer, we investigated whether subinhibitory concentrations of bronopol could enhance cell membrane permeability by assessing inner and outer membrane permeability, conducting TEM, CLSM, and measuring ROS production. NPN staining revealed significant increases in outer membrane permeability, respectively, in both donor and recipient strains treated with 2 μg/L and 20 μg/L bronopol (Figure 3A). In addition, we evaluated the cell membrane permeability using PI staining through microplate reader assays and CLSM analysis. The results showed that after pre-incubation of cells with bronopol, the fluorescence intensity increased in a concentration-dependent manner due to PI uptake and DNA binding, indicating a gradual decline in cell membrane integrity (Figure 3B and Supplementary Figure S1). In contrast to the blank control group, the bronopol-treated donor and recipient strains did not display a significant increase in fluorescence associated with ROS accumulation (Figure 3C). Concurrently, TEM images vividly revealed distinct morphological alterations in the cells induced by bronopol (Figures 3D,E). Bronopol-treated cells exhibited shrunken, roughened surfaces with distinct cytoplasmic membrane detachment, whereas control cells retained smooth, intact membranes. Collectively, these data affirm that bronopol exposure enhances cell membrane permeability, potentially facilitating the colocalization-mediated transfer of antibiotic-resistance genes.

### 3.4 Effects of bronopol on RP4 plasmid conjugation-related genes and outer membrane porins/efflux pumps

**Figure 4:**
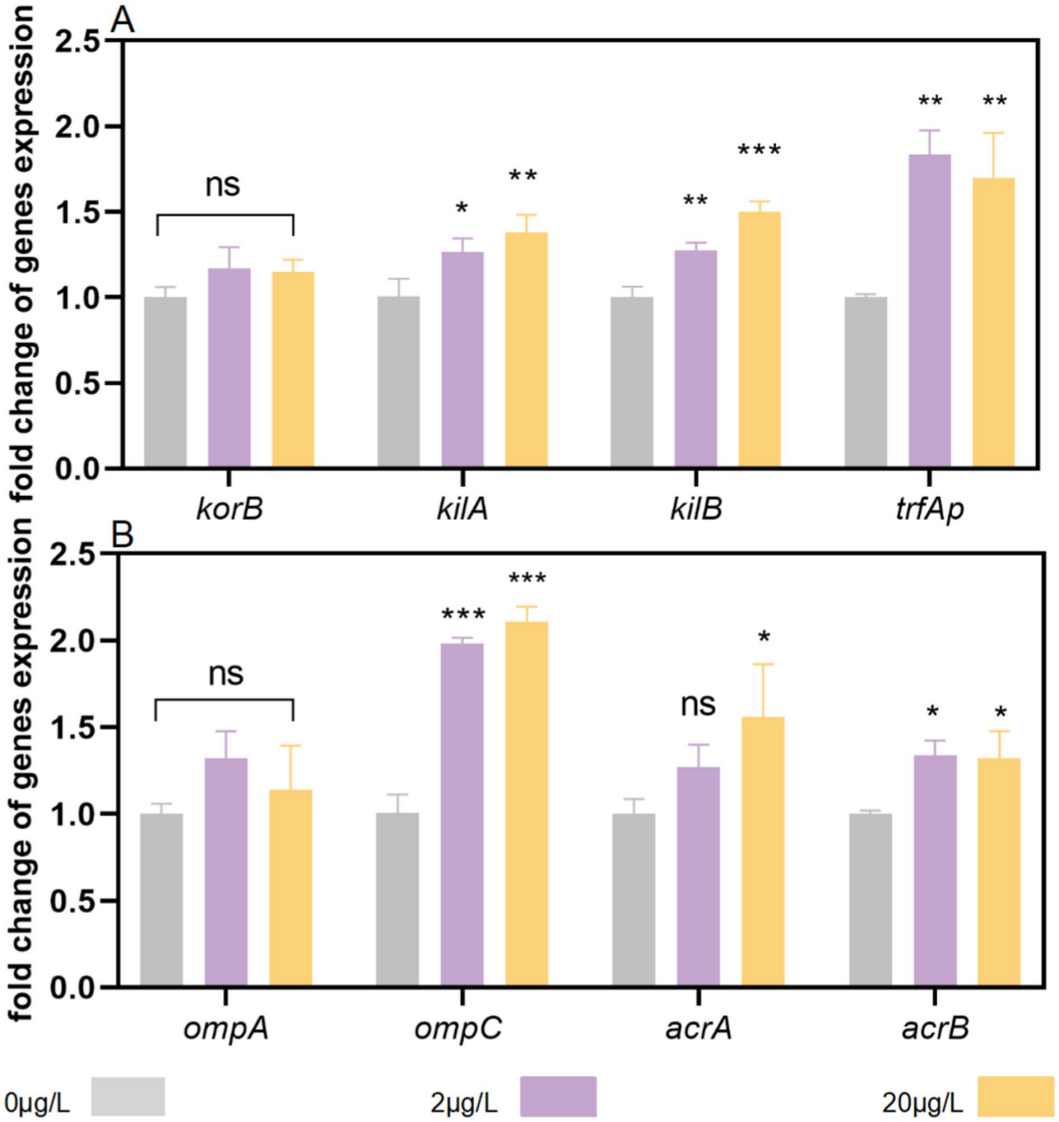
Effects of bronopol stress on the expression of genes associated with pore formation and conjugation. **(A)** The global regulation gene (*korB*), DNA transfer and replication system genes (*trfAp*), and global regulator genes (*kilA* and *kilB*). **(B)** Cell membrane porin genes (*ompA* and *ompC*) and efflux pump genes (*acrA* and *acrB*). Error bars represent the standard deviations of triplicate tests. ns, not significant, *p < 0.05, **p < 0.01; ***p < 0.001.

Plasmid conjugation is predominantly governed by three core systems: mating-pair formation, DNA transfer, and replication. To investigate bronopol’s impact on this mechanism, we analyzed transcriptional changes in genes encoding conjugation machinery components. Our results revealed significant divergences in gene expression patterns between treatment groups (Figure 4A). Following exposure to 20 μg/L bronopol during conjugation, mRNA expression levels of *trfAp*, *kilA*, and *kilB* were synergistically upregulated by 69.9, 37.5, and 49.9% compared to the control, respectively. Conversely, treatment with 2 μg/L bronopol induced no significant change in *kilA* expression, yet triggered marked increases in *trfAp* and *kilB* expression (83.5 and 27.0%, respectively). Furthermore, no significant effects of various treatment groups were detected on the global regulatory gene (*korB*). These findings suggest that bronopol promotes conjugative transfer channel formation and plasmid transmission through coordinate upregulation of global regulator genes (*kilA*/*kilB*) and the trfA promoter (*trfAp*).

Next, we investigated alterations in outer membrane porin and efflux pump gene expression at the bacterial cellular level. Among the tested genes, *ompA* expression did not differ significantly between bronopol-treated and control groups (Figure 4B). Notably, *ompC* transcription was significantly upregulated in cells exposed to both 2 μg/L and 20 μg/L bronopol. Additionally, examination of major efflux pump genes revealed a modest upregulation of *acrA* and *acrB* in bronopol-treated cells at sub-inhibitory concentrations. Collectively, these findings suggest that bronopol exposure induces coordinated increases in *ompC* membrane porins and efflux pumps.

## 4 Discussion

The misuse and overuse of antibiotics have emerged as key drivers of the global rise in AMR (Browne et al., 2021). While HGT of resistance elements across bacterial species exacerbates this crisis, the role of disinfectants in modulating HGT remains understudied. In recent years, especially against the backdrop of the COVID-19 pandemic, the usage of various disinfectants has increased sharply. Currently, the increase in the usage of disinfectants may accelerate the spread of AMR, thus posing environmental and public health risks (Hu et al., 2023). Recent research has begun to uncover disinfectants as potential facilitators of plasmid-mediated conjugation, yet most prior studies rely on laboratory strains and model plasmids (Han et al., 2019; Mantilla-Calderon et al., 2019; Lu et al., 2020). Here, we show that bronopol at environmentally relevant concentrations significantly increases CTF of RP4-7 (Supplementary Figure S2). Compared with the RP4 plasmid, clinical *IncFII(K)* plasmids typically carry multiple replicons and have more complex conjugative transfer systems (Ho et al., 2015; Rodriguez et al., 2015). The *IncFII(K)* plasmid used in this study was classified as a large plasmid type (115,297 bp, >10 kb). Fortunately, bronopol showed weak promotion of conjugative transfer of the *IncFII(K)* plasmid. Given the widespread use of bronopol, further attention should still be paid to the risk of bronopol promoting the transmission of drug-resistant plasmids in other clinical strains in the future.

The process of plasmid conjugative transfer is directly related to changes in the permeability of the cell membrane, which acts as a barrier that controls the entry and efflux of chemicals (Chen et al., 2005). Increased membrane permeability has been shown to greatly aid plasmid horizontal transfer in earlier research (Han et al., 2019; Yu et al., 2021). As a powerful membrane-disrupting agent, bronopol induces intracellular substance leakage and cell death (Lee and O'neill, 2019). Using the NPN probe to measure outer membrane permeability, the study’s findings demonstrated that bronopol treatment within the measured concentration range damaged the bacterial outer membrane. Furthermore, PI staining and laser confocal observation showed that 2 μg/L bronopol treatment also potentially increased the permeability of the plasma membrane (Figure 2A). Unlike triclosan, which relies on ROS-mediated lipid bilayer damage (Lu et al., 2018). Bronopol-induced permeability occurred independently of ROS production. This distinction highlights a novel ROS-independent pathway for disinfectant-enhanced conjugation, likely involving direct structural alterations of the bacterial cell envelope. Such membrane remodeling may facilitate plasmid translocation by creating transient pores or destabilizing the membrane barrier, as previously proposed for quaternary ammonium compounds (Liu et al., 2023).

The RP4 plasmid harbors a suite of genes essential for conjugative transfer (Miyakoshi et al., 2020; Virolle et al., 2020). In this study, bronopol exposure significantly upregulated the expression of *trfAp*, a key gene encoding the DNA transfer/replication initiator protein. This finding aligns with a recent report demonstrating concentration-dependent *trfAp* induction by glyphosate in *E. coli* (Zhang et al., 2021; Yang et al., 2022). The *kilA* and *kilB* genes are host-killing determinants inhibited by *korA* and *korB*, respectively (Goncharoff et al., 1991) . In this study, we observed the synergistic increase of the *kilA* and *kilB* genes, which is similar to the result of a previous study (Yang et al., 2022). This study suggests that the increased expression of *kilA* and *kilB* antagonizes the functions of *korA* and *korB*, leading to the release of the inhibition of RP4 transfer genes during conjugation.

Previous studies indicate that conjugation-promoting compounds induce remodeling of the bacterial outer membrane, often accompanied by upregulation of outer membrane porin genes (Rosas and Lithgow, 2022; Wu et al., 2023). However, the specific porin genes involved in this remodeling process can vary depending on the compound. For instance, the antiepileptic drug carbamazepine upregulates *ompA* and *ompN* to facilitate conjugative transfer (Wang et al., 2019). In contrast, bronopol exposure modestly induced *ompC* expression without altering *ompA* levels in this study. The increased *ompC* expression may enhance plasmid uptake by augmenting membrane permeability or creating translocation channels for RP4 transfer. Concurrent upregulation of *acrA* and *acrB*, genes that encode efflux pumps responsible for extruding antimicrobial compounds, implies a dual role for bronopol: it disrupts membrane integrity while simultaneously triggering adaptive responses in bacteria to expel the disinfectant. Collectively, these findings and data suggest that bronopol promotes plasmid dissemination through a multifaceted mechanism rather than a single pathway, involving membrane permeability enhancement, porin remodeling, efflux pump activation, as well as selectively activating plasmid-encoded transfer machinery and global regulatory networks.

Notably, this study has limitations. While bronopol enhances intergenera plasmid transfer, cross-genera validation is lacking. Additionally, although membrane permeability and conjugative gene upregulation were identified as mechanisms, other pathways, such as metabolite alterations, require further exploration. These uninvestigated aspects may involve complex interplay between disinfectant exposure and bacterial physiology, underscoring the need for broader validation and mechanistic studies to fully characterize bronopol’s impact on antibiotic resistance dissemination. A critical observation from this study is the substantial difference in bronopol’s promotion of conjugative transfer frequency between RP4-7 and *IncFII(K)*, suggesting that bronopol’s enhancing effect may exhibit plasmid specificity. Given the diversity of clinical resistance plasmids, we advocate that future studies should incorporate as many types of clinical resistance plasmids as possible to overcome the limitations of previous research that only involved RP4 plasmids.

## Data Availability

The original contributions presented in the study are included in the article/Supplementary material; further inquiries can be directed to the corresponding author.

## Supplementary material

SUPPLEMENTARY FIGURE S1 Fluorescence microscopy imaging of membrane permeability using PI staining. **(A)** PBS control; **(B)** cells treated with bronopol 2 μg/L; **(C)** cells treated with bronopol 20 μg.

SUPPLEMENTARY FIGURE S2 Frequency of RP4-7 plasmid **(A)** and *IncFII(K)* plasmid **(B)** conjugative transfer underexposure to bronopol. Significant differences between bronopol treated groups and the control were analyzed using independent-sample test: ns, not significant, **p* < 0.05; ***p* < 0.01; ****p* < 0.001.
