# Supplementary figures and images for: The ecological security risks of bronopol: a focus on antibiotic resistance gene dissemination
Source: Front Microbiol. 2025 Jul 7;16:1595833. doi: 10.3389/fmicb.2025.1595833 (PMC12277303; doi:10.3389/fmicb.2025.1595833)

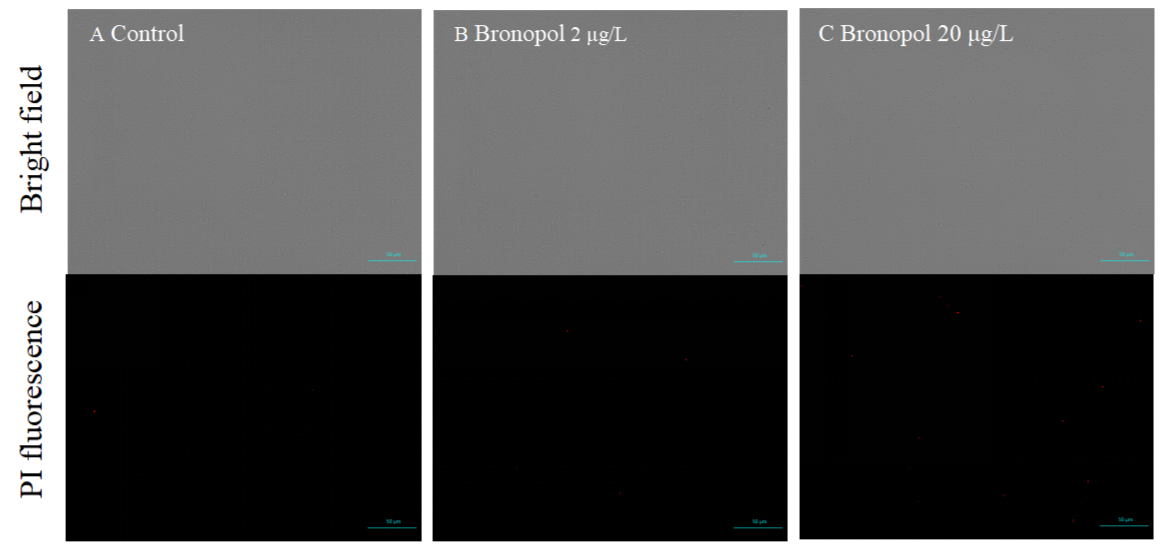

Supplement: SUPPLEMENTARY FIGURE S1 — Fluorescence microscopy imaging of membrane permeability using PI staining. (A) PBS control; (B) cells treated with bronopol 2 μg/L; (C) cells treated with bronopol 20 μg. [file Image_1.tiff]

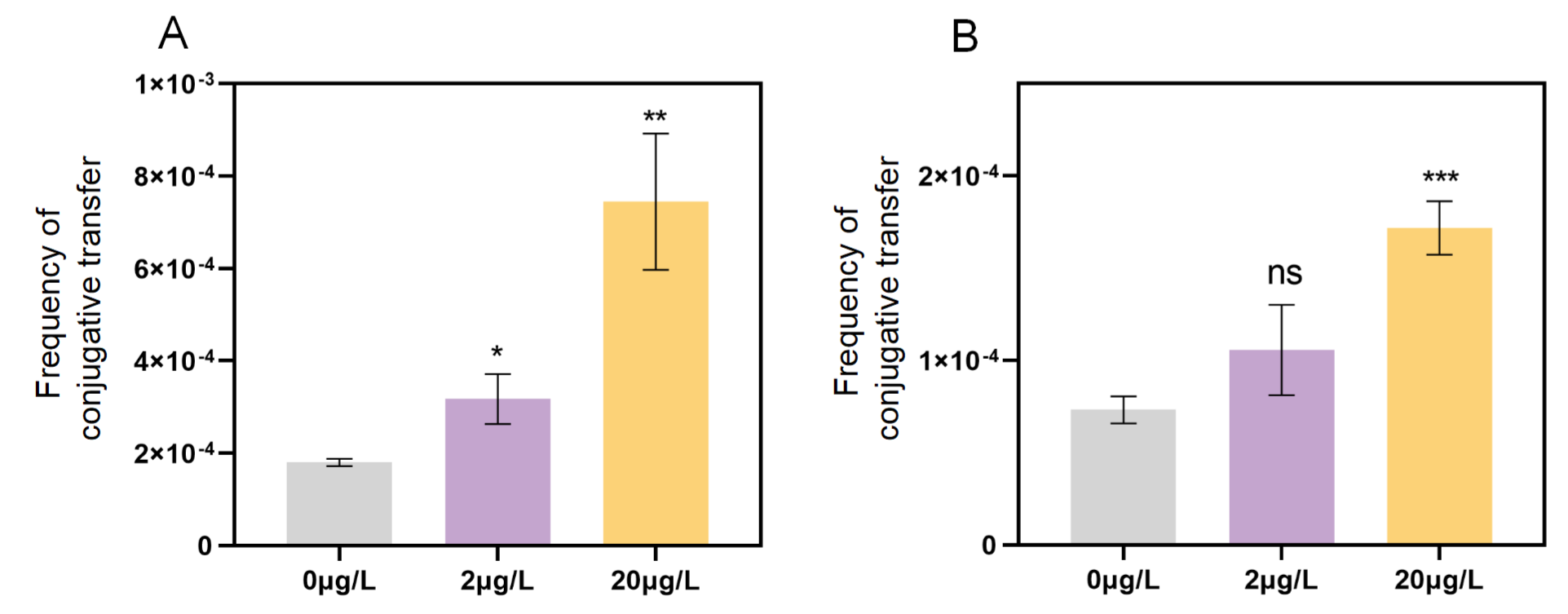

Supplement: SUPPLEMENTARY FIGURE S2 — Frequency of RP4-7 plasmid (A) and IncFII(K) plasmid (B) conjugative transfer underexposure to bronopol. Significant differences between bronopol treated groups and the control were analyzed using independent-sample test: ns, not significant, *p < 0.05; **p < 0.01; ***p < 0.001. [file Image_2.tiff]
