# Supplementary material for: The ecological security risks of bronopol: a focus on antibiotic resistance gene dissemination
Source: Front Microbiol. 2025 Jul 7;16:1595833. doi: 10.3389/fmicb.2025.1595833 (PMC12277303; doi:10.3389/fmicb.2025.1595833)
Supplement: Supplementary file 3 [file Table_1.docx]

Table S1.Primers used in this study

| Gene | Primer | Sequence(5’-3’) | Source |
| --- | --- | --- | --- |
| *korA* | FW | GACGATGTACGCCTGATGTT | ^1^ |
| *korA* | RV | CTTACCGAAAGGCAGTTCC |  |
| *korB* | FW | TATCGCCTTCTTCTTGCC |  |
| *korB* | RV | GACTACAACGAAGCCGACC |  |
| *trfAp* | FW | CGCAATATCGAACAAGGAA |  |
| *trfAp* | RV | GAACTATGACGACCAGAAGC |  |
| *kilA* | FW | ACCAGGTAGGCTTTTCCG |  |
| *kilA* | RV | ATTCCCTCAACGAGCTGTC |  |
| *kilB* | FW | AGAACGCGTCCAATACC |  |
| *kilB* | RV | TCCAGGCCATCAACAGATC |  |
| *16s rRNA* | FW | CCTACGGGAGGCAGCAG |  |
| *16s rRNA* | RV | ATTACCGCGGCTGCTGG |  |
| *ompA* | FW | TGAGCCTGGGTGTTTCCTA |  |
| *ompA* | RV | CAGAGCAGCCTGACCTTCC |  |
| *ompC* | FW | GGCGACAAAAGCACAGAA |  |
| *ompC* | RV | AAGTAGTAGGTAGCACCAACATCA |  |
| *acrA* | FW | AGAAGTTCCGTCTCAAGTTAGC |  |
| *acrA* | RV | CACCTTTCGCACTGTCGTAT |  |
| *acrB* | FW | TTGCGTTCGTTTCCTTGA |  |
| *acrB* | RV | GGTTGCAGTACCCAGTTCC |  |

1. Wu, J.; Zhou, J. H.; Liu, D. F.; Wu, J.; He, R. L.; Cheng, Z. H.; Li, H. H.; Li, W. W., Phthalates Promote Dissemination of Antibiotic Resistance Genes: An Overlooked Environmental Risk. *Environ Sci Technol* **2023,** *57* (17), 6876-6887.
